# Current global research on mandibular defect: A bibliometric analysis from 2001 to 2021

**DOI:** 10.3389/fbioe.2023.1061567

**Published:** 2023-03-23

**Authors:** Yongdi Li, Duchenhui Li, Zhenglong Tang, Dongxiang Wang, Zhishan Yang, Yiheng Liu

**Affiliations:** ^1^ School of Basic Sciences, Guizhou Medical University, Guiyang, China; ^2^ School of Stomatology, Guizhou Medical University, Guiyang, China

**Keywords:** mandibular defect, biblionetric, computer-aided design, 3D printing, bone tissue engineering

## Abstract

**Background:** Mandibular defects can result from congenital deformities, trauma, tumor resection, and osteomyelitis. The shape was irregular because the lower jaw was radians. This involves teeth and jaw functions; therefore, the difficulty of bone repair is greater than that in other body parts. Several standard treatments are available, but they result in various problems, such as difficulties in skin flap transplantation and possible zone dysfunction, artificial material boneless combining ability, and a long treatment period. This study aimed to introduce the present status of research on mandibular defects to analyze the current introduction and predict future research trends through a bibliometric study.

**Methods:** From 2001 to 2021, publications on mandibular defects were collected for bibliometric visualization using VOSviewer, CiteSpace, and Scimago Graphica software based on the Web of Science Core Collection.

**Results:** This study analyzed 4,377 articles, including 1,080 published in the United States, 563 in China, and 359 in Germany, with an increase in the number of articles published over the past 20 years. Wikesjoe and Ulf Mai E had the most publications (*p* = 36) and citations (citations = 1,553). Shanghai Jiaotong University published the highest number of papers among the research institutions (*p* = 88). The most productive journal was Journal of Oral and Maxillofacial Surgery, and the cited literature was primarily classified as dentistry, dermatology, and surgery. Cluster Analysis of Co-occurrence Keywords revealed that highest number of core words were mandibular defects, mandibular reconstruction, and bone regeneration. The highest cited words were head and neck cancer, accuracy, and osteogenic differentiation. High-frequency terms of Cluster Analysis of References were osteosynthesis plate, tissue engineering, and rapid distraction rate.

**Conclusion:** Over the past 20 years, the number of studies on mandibular defects has gradually increased. New surgical procedures are increasingly being used in clinical practice. Current frontier topics mainly focus on areas such as computer-aided design, 3D printing of osteotomy and reconstruction guide plates, virtual surgical planning, and bone tissue engineering.

## Introduction

Mandibular defects can result from congenital deformities, trauma, tumor resection, and osteomyelitis (Mitrea et al., 2020). These defects cause severe external deformities and dysfunction in patients due to their special anatomical position, causing facial deformities, partial jaw, partial collapse, and other severe facial deformities, including chewing and language dysfunction, and affect the physical and mental health of patients, seriously reducing their quality of life (Kumar et al., 2016).

The primary treatment methods for mandibular defects include free bone transplantation, vascularized free bone flap transplantation, stem cell tissue engineering, and distraction osteogenesis (Tang et al., 2016; Blumberg et al., 2019; Jin et al., 2019; Tee and Sun, 2020). The goal of treatment is to reconstruct the shape and function of the mandible and restore the patient’s facial shape, swallowing, and language function. The mandible is more difficult to repair than other parts of the bone because of its radians, irregular shapes, dentition, and oral function. Mandibular defects greatly affect jaw function. There are various insolvable problems, such as free bone graft infection, bone resorption, and flap transplantation. It may cause donor site dysfunction; artificial materials have no bone-binding ability, and the distraction osteogenesis treatment cycle is long (Hopper et al., 2020; Shao et al., 2020; Li et al., 2022; Shin et al., 2022; Wu et al., 2022). Therefore, there is no ideal method for treating mandibular defects. Recently, numerous studies have reported the application of three-dimensional (3D) printing technology, biomaterials, stem cell tissue engineering technology, and rapid distraction osteogenesis in mandibular defects, providing new ideas for treating mandibular defects (Tang et al., 2016; Gjerde et al., 2018; Lu et al., 2020; Kang et al., 2021; Li et al., 2022; Steffen et al., 2023). There is no global research that tests and predicts the research frontier of mandibular defects despite the abundance of literature on this topic. Therefore, we aim to conduct a bibliometric analysis to provide a scientific reference for scholars.

Citespace and VOSviewer (Synnestvedt et al., 2005; van Eck and Waltman, 2010) were used by bibliometric (Chen, 2004) analysis to track disciplinary development and explore the application of knowledge in specific medical fields (Zhou et al., 2022). Presently, the literature on mandibular defects has not been used to summarize the development trend. Therefore, this study aimed to visually analyze the research hotspots of mandibular defects and predict potential development trends using bibliometric methods.

## Methods

**FIGURE 1:**
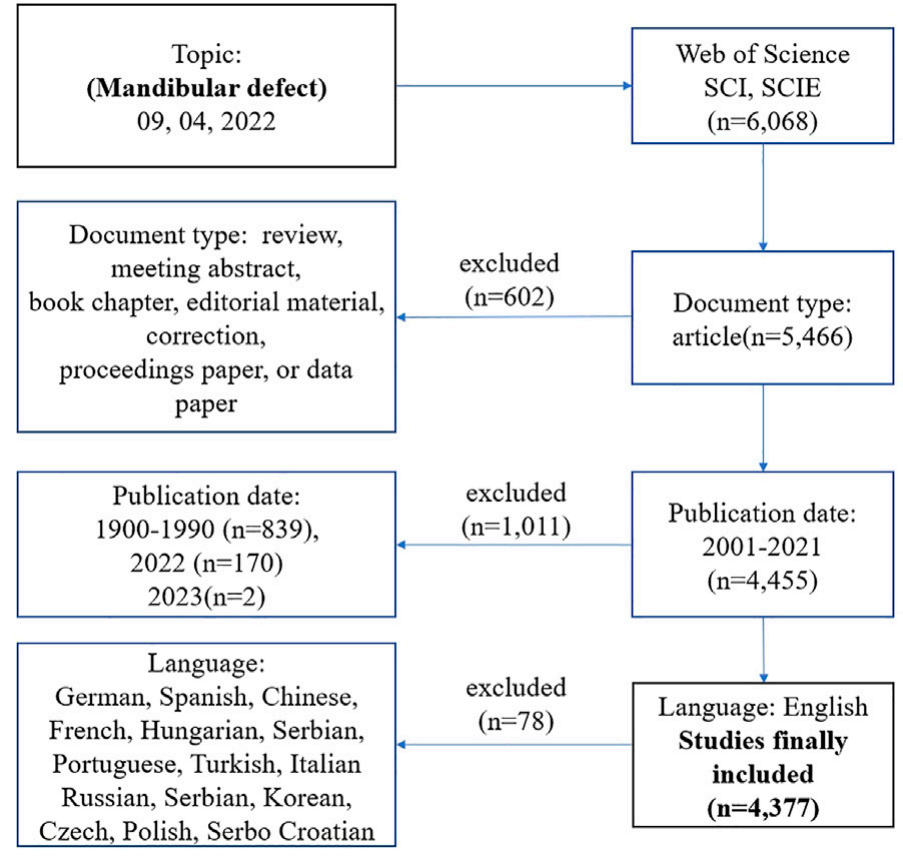
Frame flow diagram of mandibular defect search strategy from 2001 to 2021 based on Web of Science.

All data were collected from Science Citation Index Expanded and Social Sciences Citation Index databases of Web of Science Core Collection (WoSCC). The final suitable results were chosen using different search strategies, including various publication periods or topics. Any divergences were settled through consultation with external specialists to reach a consensus. The retrieval strategy was as follows: (topic = mandibular defect), (type = article), (year published = 2001–2021), and (Language = English). The retrieval date was September 9, 2022, by two researchers (Figure 1 **)**.

VOSviewer 1.6.16 and CiteSpace 6.1. R3 were applied to identify co-cited articles, keywords, countries, institutions, journals, authors, and reference bursts. The H-index, impact factor, and category quartiles were obtained from the Journal Citation Report 2021. The data on publications, citations, and polynomial trend lines were analyzed using Excel software. CiteSpace software was used to construct the hotspots and knowledge base map of mandibular defect research and to detect centrality. VOSviewer was used to analyze the authorship and co-occurrence of keywords, visualizing hotspots in the mandibular defect field by clustering them with different colors. Scimago Graphica 1.0.18 was used to visualize the collaborative relationships between countries/regions.

## Results

### Literature development trends

**FIGURE 2:**
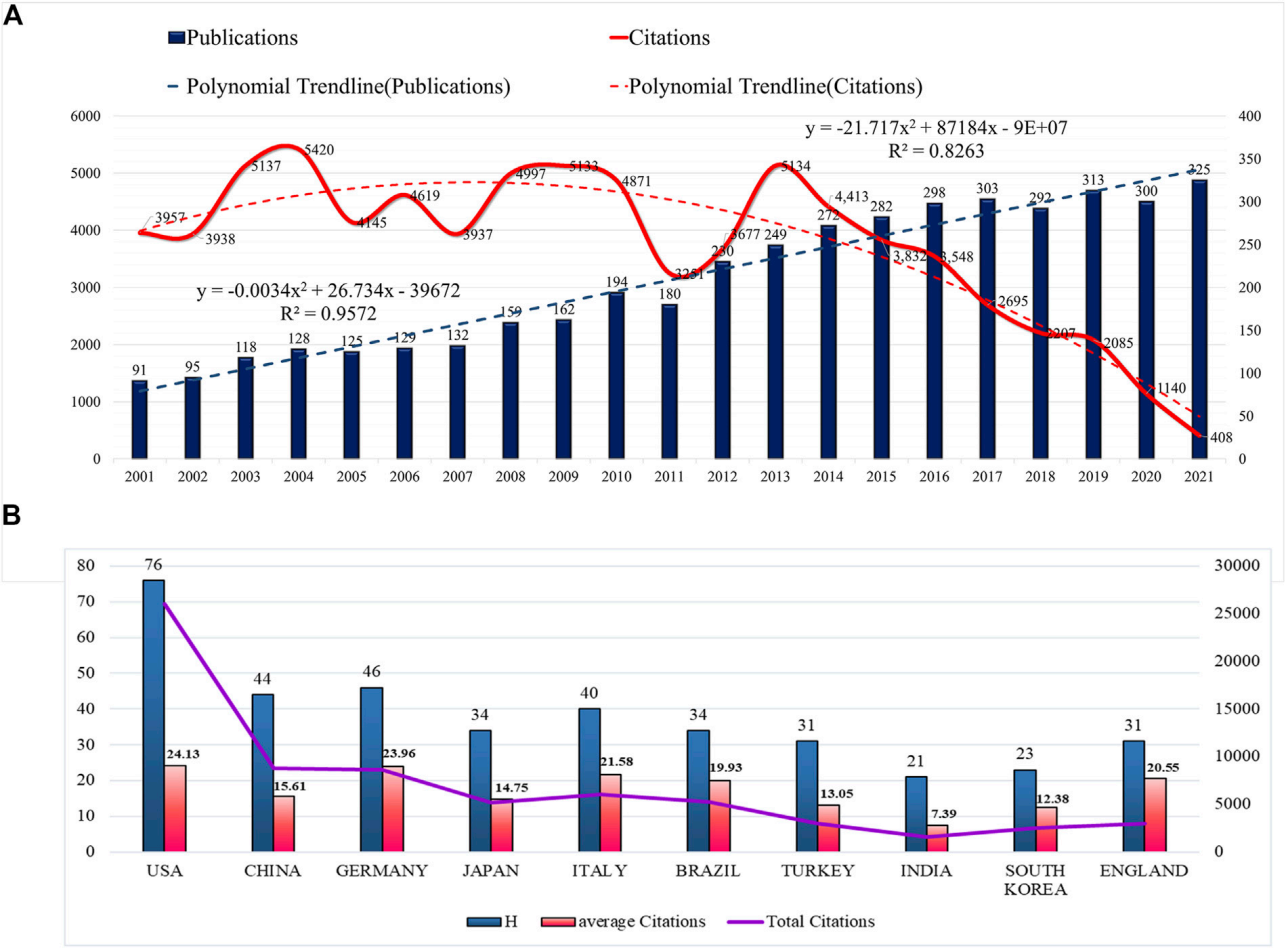
Trends in publications and citations of mandibular defect research. **(A)** The annual trends of global publications and citations. **(B)** H-index, average citations (citations per article), and total citations of the top ten countries.

According to WoSCC, 4,377 articles on mandibular defects were published between 2001 and 2021. The total number of published papers has demonstrated an upward trend over the past 20 years (Figure 2A). From 2001 onwards, the number of papers on mandibular defects increased with continuous growth until 2021, except for 2005, 2009, 2018, and 2020. The number of publications in 2021 was over three times that in 2001. Furthermore, the highest citations occurred in 2004, 2009, and 2013. It began to decrease after 2013. The linear fitting of articles on mandibular defects revealed a significant correlation (R^2^ = 0.8263) between the year and citations, attracting widespread attention from scientists in the mandibular defect field worldwide.

### Geographic distribution

**FIGURE 3:**
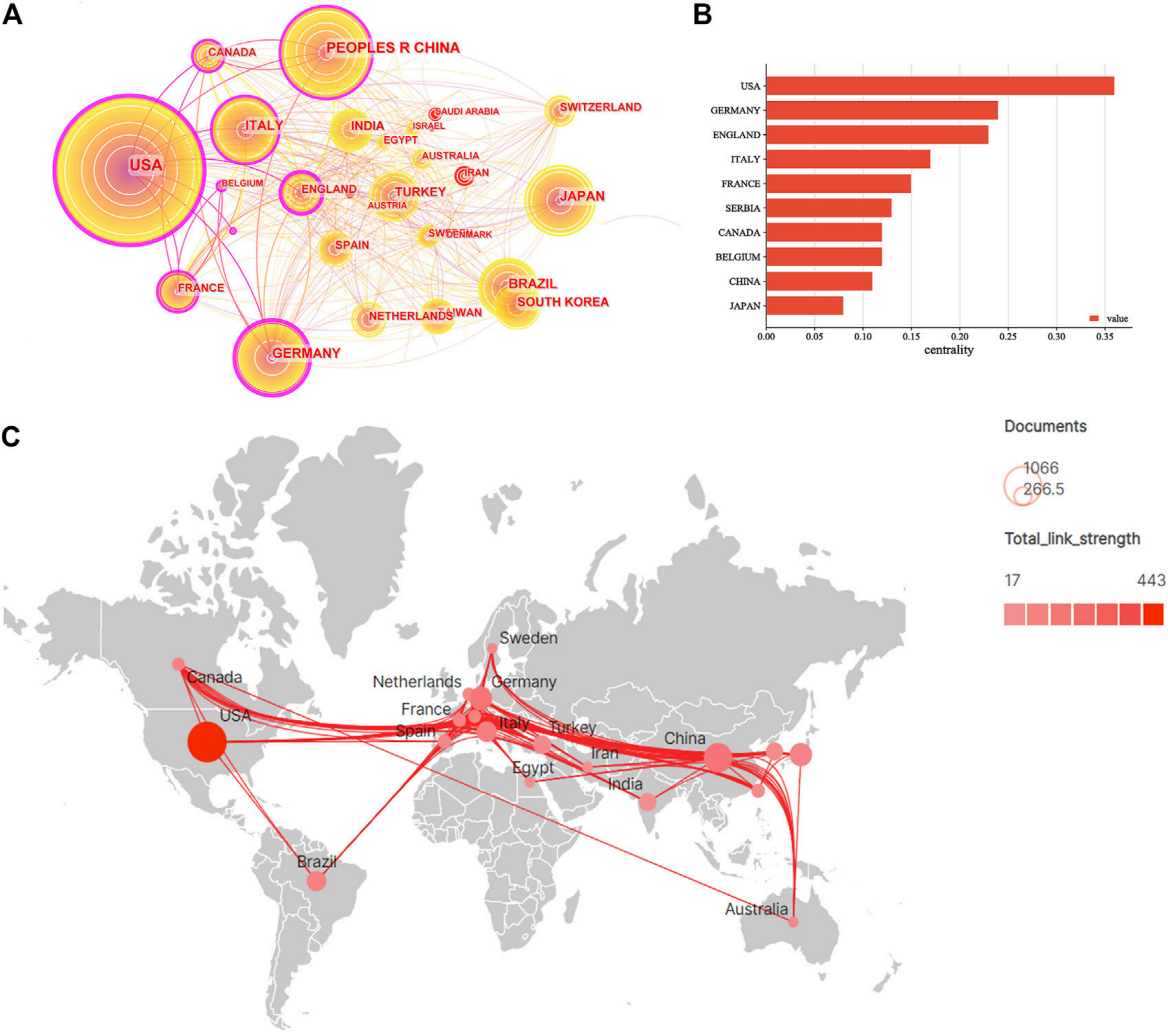
Visualization map of countries/regions involved of mandibular defect research. **(A)** Collaboration network of countries of CiteSpace. *N* = 92, *E* = 388. (*N* represents the number of network nodes. *E* represents the number of connections). **(B)** The centrality of countries/regions of mandibular defect research. **(C)** Collaborative relationships in countries/regions.

The literature was distributed among 92 countries/regions and 3,716 institutions. The top five countries of publications (P) were the United States (*p* = 1,080, 24.67%), China (*p* = 563, 12.86%), Germany (*p* = 359, 8.20%), Japan (*p* = 352, 8.04%), and Italy (*p* = 282, 6.44%), in the top 10 countries (Table 1). Additionally, an effort was made to build the countries’ annual national publications and citations (Figure 2B). The top three countries with the highest mean citations were the United States (mean citations = 24.13), Germany (mean citations = 23.96), and Italy (mean citations = 21.58). The top three countries in the H-index were the United States (H = 76), Germany (H = 46), and China (H = 44). Among them, the United States, Germany, England, Italy, France, Serbia, Canada, Belgium, and China were core nodes (centrality > 0.1), marked with a purple circle (Figures 3A, B). The above results demonstrated that mandibular defects received widespread attention from global scholars, with the United States and Germany being the leading contributors. Furthermore, the United States represented closer cooperation in mandibular defect research (Figure 3C).

**TABLE 1:** Top ten countries by publications, H-index, and citations of mandibular defect research.

Rank	Countries/regions	Publications	(% of 4,377)	H-index	Total citations	Average citations
1st	United States	1080	24.67	76	26,058	24.13
2nd	CHINA	563	12.86	44	8,791	15.61
3rd	GERMANY	359	8.20	46	8,601	23.96
4th	JAPAN	352	8.04	34	5,191	14.75
5th	ITALY	282	6.44	40	6,085	21.58
6th	BRAZIL	265	6.05	34	5,282	19.93
7th	TURKEY	227	5.19	31	2,962	13.05
8th	INDIA	217	4.96	21	1,604	7.39
9th	SOUTH KOREA	207	4.73	23	2,563	12.38
10th	ENGLAND	146	3.33	31	3,000	20.55

**FIGURE 4:**
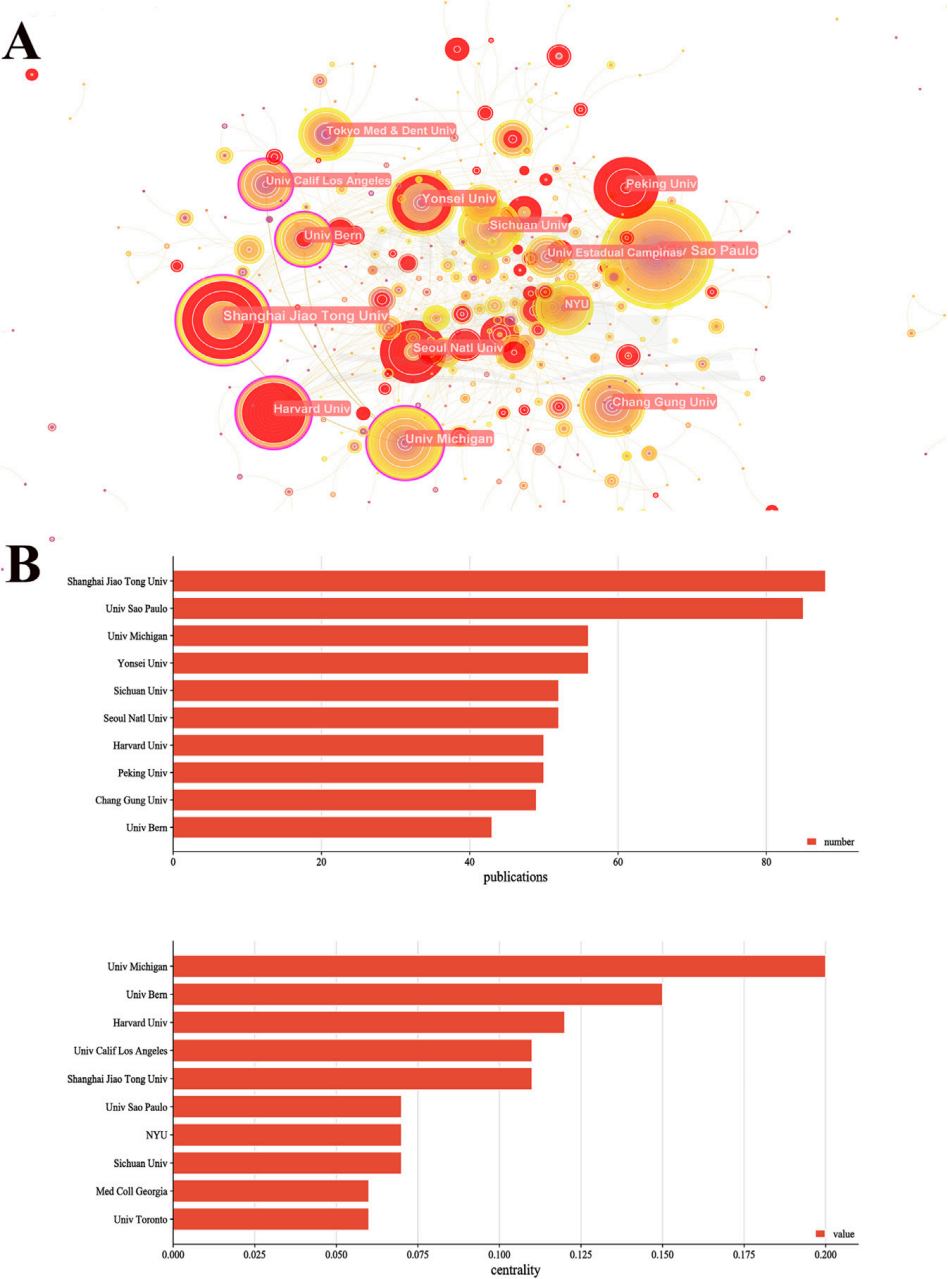
Collaboration network of institutions of CiteSpace. **(A)** Visualization map of institutions. *N* = 598, *E* = 678. (*N* represents the number of network nodes. *E* represents the number of connections). **(B)** The publications and centrality of institutions of mandibular defect research.

Shanghai Jiaotong University (P = 88), University of São Paulo (P = 85), University of Michigan (P = 56), Yosei University (P = 56), and Sichuan University (P = 52) had the highest number of publications, most of which originated from the United States and China (Figures 4A, B

). Additionally, the University of Michigan, the University of Bern, Harvard University, the University of California, Los Angeles, and Shanghai Jiaotong University were the core nodes with high centrality (centrality > 0.1) (Figure 4C); Most of them originated from the United States. These results suggest that institutions in the United States and China are the main research forces. However, centrality values were still low, and global cooperation should be strengthened in the mandibular defect research field.

### Contributions of authors and Co-cited authors

**FIGURE 5:**
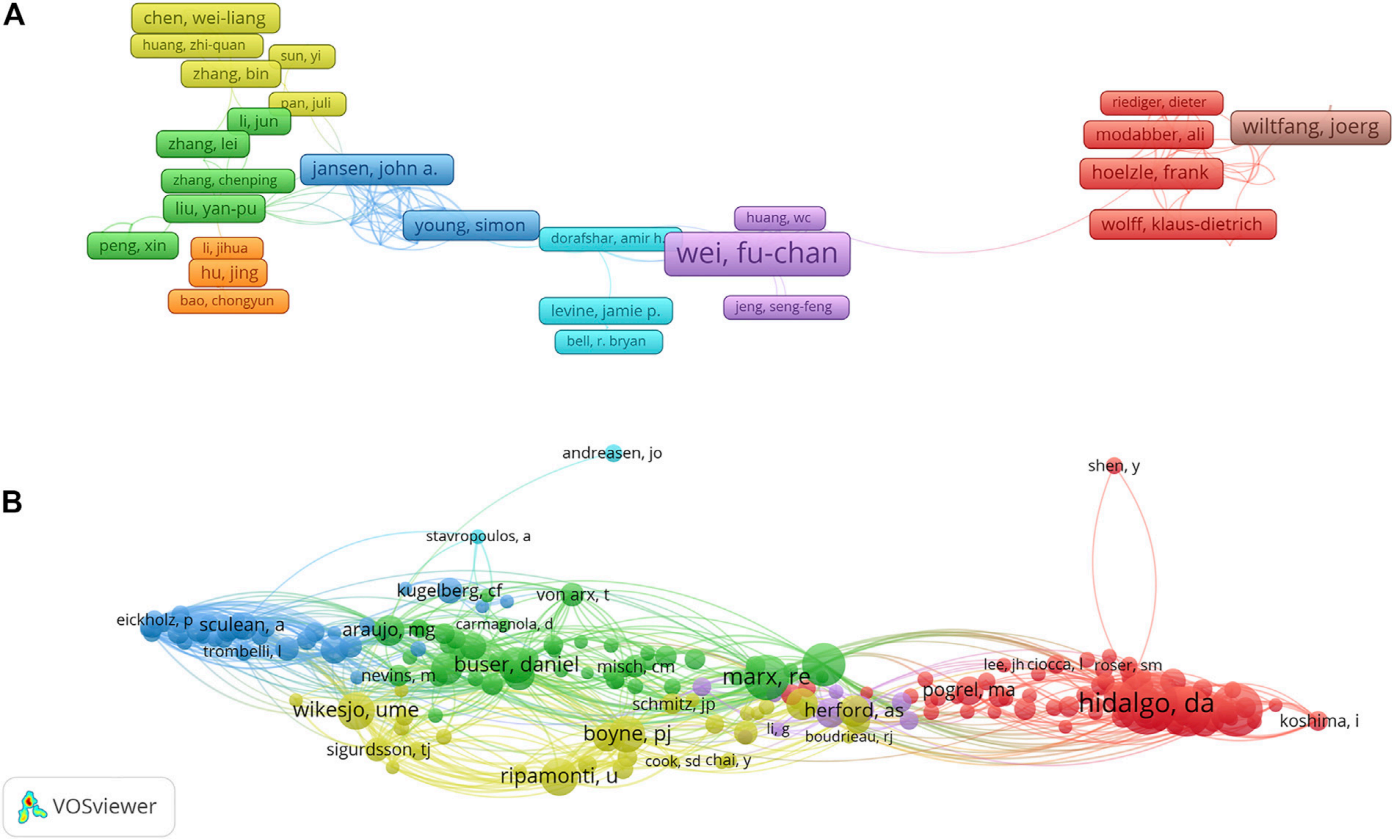
VOSviewer visualization map of authors and co-cited authors devoted to mandibular defect research. **(A)** Cooperation network of authors. Of the 18, 209 authors, 183 had published at least six documents. **(B)** Co-citation network of authors. Of the 46,363 co-cited authors, 200 had at least 50 citations.

A total of 18,209 authors were selected for mandibular defect research. Wikesjoe and Ulf (*p* = 36) were the most productive authors from the United States, followed by Wei, Fu-Chan (*p* = 35), Nociti Junior, Francisco (*p* = 26), Sallum, E. A. (*p* = 26), and Choi, Seong-Ho. Years (*p* = 23), respectively **(** Table 2 **)**. The top three most-cited authors were Wikesjoe, Ulf M. E (citations = 1,553), Wei, Fu-Chan (citations = 1,468), and Wiltfang and Joerg (citations = 1,089). In the cooperation network of the authors, Wei and Fu-Chan cooperated closely with Huang, WC, and Jeng, Seng-Feng **(** Figure 5A **)**. Furthermore, the H-index of Wikesjoe, Ulf M. E (H = 24) was the highest and cooperated closely with Sigurdsson TJ in the co-citation network **(** Figure 5B **)**.

**TABLE 2:** Top ten authors distributed by publications of mandibular defect research.

Rank	Cited author	Publications	Total Citations	Average Citations	H-index	Country/Region
1st	Wikesjoe, Ulf M. E	36	1,553	43.14	24	United States
2nd	Wei, Fu-Chan	35	1,468	41.94	22	TAIWAN
3rd	Nociti Junior, Francisco	26	635	24.42	16	BRAZIL
4th	Sallum, E. A.	26	427	18.57	13	BRAZIL
5th	Choi, Seong-Ho	23	469	20.39	11	SOUTH KOREA
6th	Jansen, John A.	21	841	40.05	17	NETHERLANDS
7th	Wiltfang, Joerg	20	1,089	54.45	12	GERMANY
8th	Hoelzle, Frank	20	377	18.85	12	GERMANY
9th	Casati, Marcio Z	19	339	17.84	11	BRAZIL
10th	Wolff, Klaus-Dietrich	18	465	25.83	13	GERMANY

### Journal analysis

**FIGURE 6:**
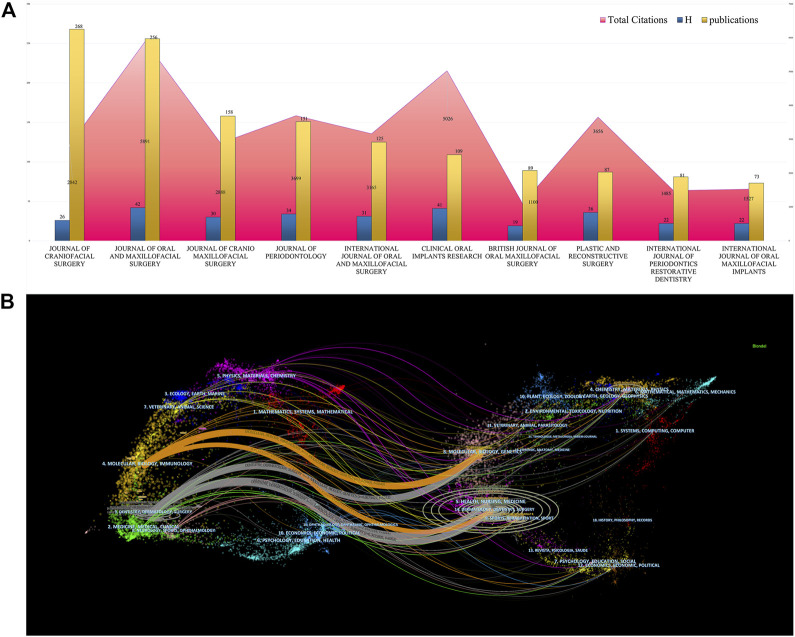
Journals analysis of mandibular defect research. **(A)** The publications, total citations, and H-index of journals of mandibular defect research. **(B)** The dual-map overlay of journals of mandibular defect research. The left circles were targeted literature while right circles were source literature.

A total of 738 journals were obtained, 12 of which contained more than 50 articles. The top three prolific and most cited journals were the *Journal of oral and maxillofacial surgery* (IF = 2.136), *Clinical Oral Implants Research* (IF = 5.021), and *Plastic and reconstructive surgery* (IF = 5.169) **(** Figure 6A **).**

The dual-map overlays depict effective collaboration between publications, citing references in diverse fields **(** Figure 6B **)** and papers primarily centered on dentistry, dermatology, surgery, molecular biology, and immunology. Publications citing the fields of dentistry, dermatology, surgery, health, nursing, and medicine have played crucial roles in mandibular defect research.

### Cluster analysis of Co-occurrence keyword

**FIGURE 7:**
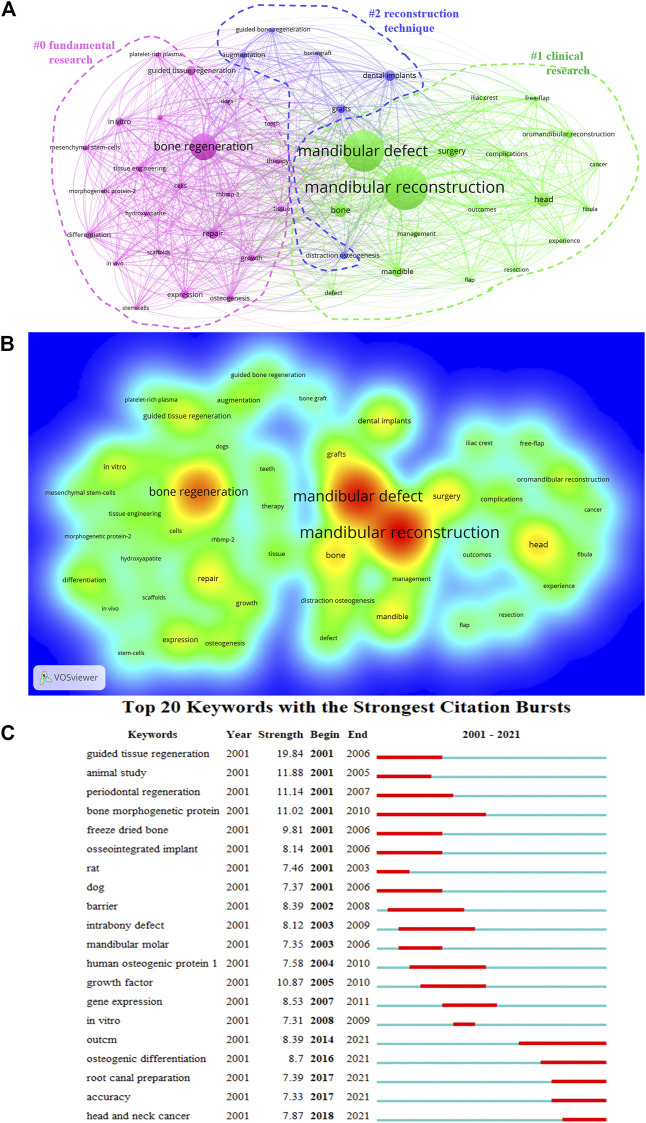
Analysis of all keywords of mandibular defect research. **(A)** VOSviewer visualization map of co-occurring keywords. Of the 11,881 keywords, 50 had at least 80 co-occurrences. **(B)** The density map of keywords. **(C)** Top 20 keywords with the strongest citation burst.

VOSviewer then created a map containing 50 terms (11,881 in total), with at least 80 appearances per term, including #0 fundamental research, #1 clinical research, and #2 reconstruction technique **(** Figure 7A **)**. The frequency of the keywords was constructed to ascertain their density, mandibular defect, mandibular reconstruction, and bone regeneration occupying a core part of the mandibular defect field **(** Figure 7B **)**. The top 20 keywords with the strongest citation bursts represented the frontiers in the mandibular defect field. The burst strength ranged from 7.31 to 19.84, suggesting that head and neck cancer, accuracy, and root canal preparation were frontiers in the mandibular defect field (Figure 7C).

### Analysis of highly cited and Co-cited articles

Table 3 displays the top 10 highly cited literature on mandibular defects. These studies have mainly focused on materials and surgery. An article published in the Journal of Oral and Maxillofacial Surgery demonstrated that virtual surgery could increase the accuracy of mandibular reconstruction and provide a future research direction for precise mandibular reconstruction. In another article published in Biomaterials, porous β-tricalcium phosphate loaded with bone marrow mesenchymal stem cells was used to repair mandibular defects by tissue engineering, laying the foundation for future bone tissue engineering research.

**TABLE 3:** Top ten highly cited literature of mandibular defect research.

Rank	Citations	Author	Title	Source	IF	Year	DOI
1st	530	Warnke, PH	Growth and transplantation of a custom vascularised bone graft in a man	*Lancet*	202.731	2004	10.1016/S0140-6736(04)16935-3
2nd	304	d'Aquino, R	Human mandible bone defect repair by the grafting of dental pulp stem/progenitor cells and collagen sponge biocomplexes	*European Cells and Materials*	4.325	2009	10.22203/eCM.v018a07
3rd	306	Grayson, WL	Engineering anatomically shaped human bone grafts	*Proceedings of the national academy of sciences of the united states of america*	12.779	2010	10.1073/pnas.0905439106
4th	290	Hidalgo, DA	Free-flap mandibular reconstruction: A 10-year follow-up study	*Plastic and reconstructive surgery*	5.169	2002	10.1097/00006534-200208000-00010
5th	269	Jensen, SS	Bone healing and graft resorption of autograft, anorganic bovine bone and beta-tricalcium phosphate. A histologic and histomorphometric study in the mandibles of minipigs	*Clinical oral implants research*	5.021	2006	10.1111/j.1600-0501.2005.01257.x
6th	263	Roser, SM	The Accuracy of Virtual Surgical Planning in Free Fibula Mandibular Reconstruction: Comparison of Planned and Final Results	*Journal of oral and maxillofacial surgery*	2.136	2010	10.1016/j.joms. 2010.06.177
7th	216	Leucht, P	Embryonic origin and Hox status determine progenitor cell fate during adult bone regeneration	*Development*	6.862	2008	10.1242/dev.023788
8th	210	Okay, DJ	Prosthodontic guidelines for surgical reconstruction of the maxilla: A classification system of defects	*Journal of prosthetic dentistry*	4.148	2001	10.1067/mpr. 2001.119524
9th	220	Yuan, J	Repair of canine mandibular bone defects with bone marrow stromal cells and porous beta-tricalcium phosphate	*Biomaterials*	15.304	2007	10.1016/j.biomaterials. 2006.10.015
10th	223	Park, J	Bone regeneration in critical size defects by cell-mediated BMP-2 gene transfer: a comparison of adenoviral vectors and liposomes	*Gene therapy*	4.184	2003	10.1038/sj.gt.3301960

The influence of time and co-citation was considered in citation analysis to avoid the effect of publication year (Table 4), which mainly focused on computer-assisted and virtual surgical plans. Most of these researches indicate the accuracy of mandibular reconstruction. The top ten co-citation references demonstrated that computer-assisted technology has recently become important.

**TABLE 4:** Top ten co-citation references of mandibular defect research.

Rank	Frequency	Title	Source	IF(JCR 2020)	Year	DOI
1st	56	A new classification for mandibular defects after oncological resection	*Lancet oncology*	54.433	2016	10.1016/S1470-2045(15)00310-1
2nd	30	Mandibular reconstruction using computer-aided design and computer-aided manufacturing: an analysis of surgical result	*Journal of oral and maxillofacial surgery*	2.136	2013	10.1016/j.joen. 2012.11.045
3rd	30	Mandibular Reconstruction: Overview	*Journal of oral and maxillofacial surgery*	2.136	2016	10.1007/s12663-015-0766-5
4th	30	Mandibular reconstruction with vascularized bone flaps: a systematic review over 25 years	*British Journal of Oral and Maxillofacial surgery*	2.018	2017	10.1016/j.bjoms. 2016.12.010
5th	28	Computer-assisted design and rapid prototype modeling in microvascular mandible reconstruction	*Laryngoscope*	2.970	2013	10.1002/lary.23717
6th	27	Contemporary reconstruction of the mandible	*Oral oncology*	5.972	2010	10.1016/j.oraloncology. 2009.11.006
7th	27	The accuracy of virtual surgical planning in free fibula mandibular reconstrction: comparison of planned and final results	*Journal of oral and maxillofacial surgery*	2.136	2010	10.1016/j.joms. 2010.06.177
8th	23	Use of virtual surgery and stereolithography-guided osteotomy for mandibular reconstruction with the free fibula	*Plastic and reconstructive surgery*	5.169	2011	10.1097/PRS.0b013e31822b6723
9th	25	Mandibular reconstruction in adults: a review	*International journal of oral and maxillofacial surgery*	2.986	2008	10.1016/j.ijom. 2008.03.002
10th	24	Three-dimensional virtual surgery accuracy for free fibula mandibular reconstruction: planned *versus* actual results	*Journal of oral and maxillofacial surgery*	2.136	2014	10.1016/j.joms. 2014.07.024

### Cluster Analysis of References

**FIGURE 8:**
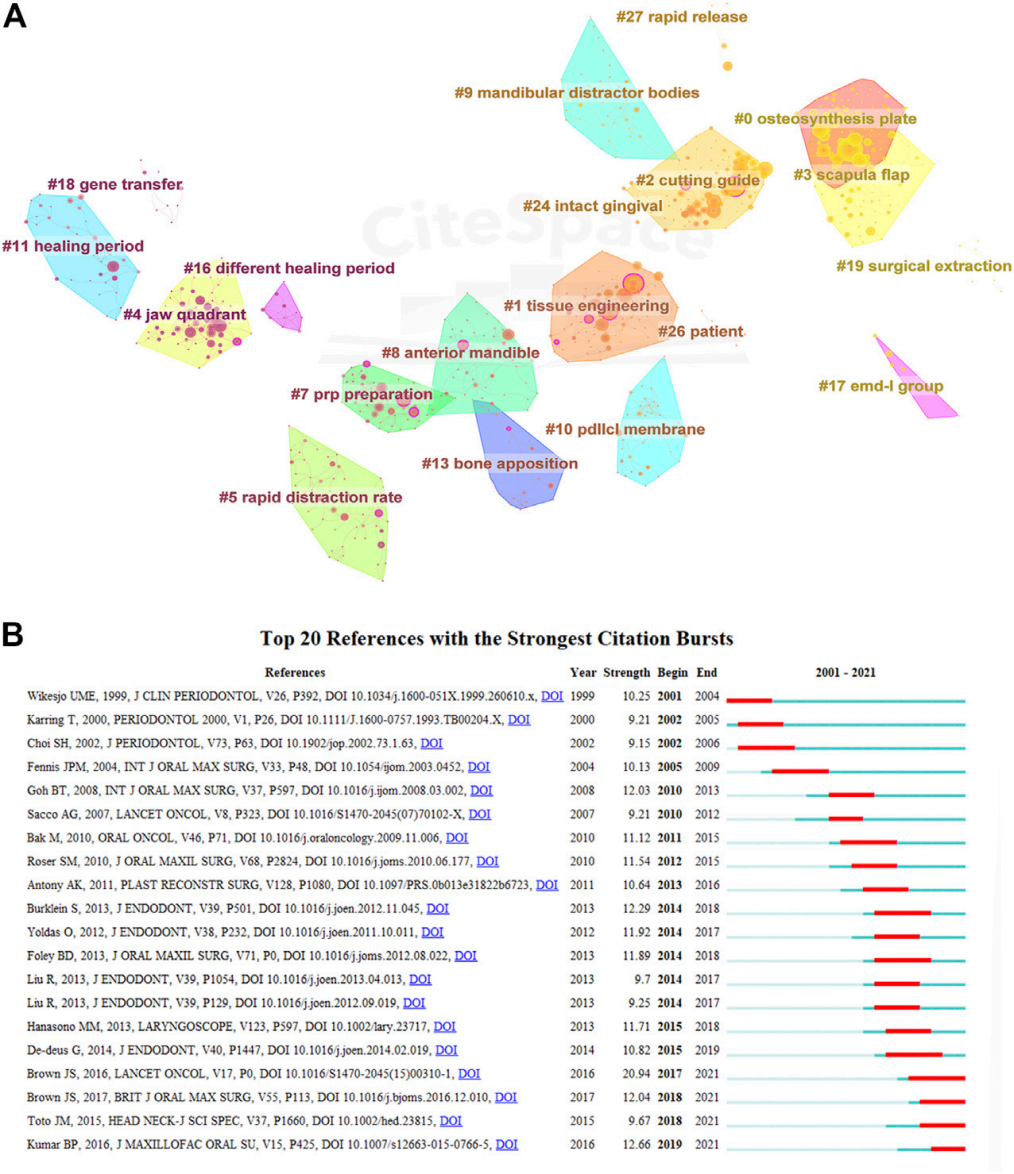
Cluster analysis of most commonly cited references of mandibular defect research. **(A)** Co-citation network of references (modularity Q = 0.8597, Sihouette S = 0.9333). **(B)** Top 20 references with the strongest citation burst.

The high-frequency terms in mandibular defect research were clustered in the same color (Figure 8A), mainly including the #0 osteosynthesis plate, #1 tissue engineering, and #5 rapid distraction rate. In our study, the modularity Q = 0.8597 and the mean S of the clusters was 0.9333, indicating that the clusters were convincing.

The top 20 references with the strongest citation burst strength represented the frontiers in the nephrotoxicity field, ranging from 9.15 to 20.94 (Figure 8B). In the last 5 years, citation bursts have indicated that scientists have mainly focused on using flaps or tissue engineering to rebuild the mandible.

## Discussion

To date, this study systematically reviewed the literature on global mandibular defects published 22 years ago. The lower jaw is an integral part of the facial bones because it is the only movable facial bone, and its repair effect and the patient’s face and voice, which are closely related to the social function and oral function, are more difficult to reconstruct than other parts of the body’s bone reconstruction of mandibular reconstruction. This study used bibliometrics to analyze articles on mandibular defects worldwide from 2001 to 2021 to provide a research introduction and hot spot for scholars and a reference for future research.

### General data

Since 1992, the number of publications on mandibular defects has increased steadily. In 2021, the number of publications reached 325, the highest value in the past 22 years. Regarding the number of citations, the United States, Germany, and China were the most cited countries. The H index of the United States, China, and Germany was the highest. These three countries have contributed the most to the study of mandibular defects. The number of citations for articles published in 2003-2004, 2008-2010, and 2013 peaked, whereas the number of citations published after 2014 decreased annually. This could be because distraction osteogenesis technology and various vascularized flaps were used as new methods for mandibular repair in 2003-2004. There are numerous reports of BMP-2 in the literature (Kuriakose et al., 2003; Park et al., 2003; Tang et al., 2016). From 2008 to 2010, the main research direction in the literature changed to stem cell and tissue engineering (d'Aquino et al., 2009; Grayson et al., 2010). In 2013, research on bone tissue engineering and biomaterials gradually increased. Simultaneously, virtual surgery and computer-aided design have gradually become mainstream surgical methods for repairing mandibular defects (Fricain et al., 2013; Hanasono and Skoracki, 2013; Wang et al., 2013). Technical innovation and research content have been milestones in mandibular defect repair in recent years, but after 2013, the number of citations has gradually decreased. Recently, there have been no revolutionary breakthroughs in basic and clinical research on mandibular defects. The publication time is short, leading to a steady decline in citations. Analysis from the perspective of different countries, including the United States and Germany, articles published recently on surgical reconstruction of implant materials, biological engineering, cell factor research, literature published in China mainly by computer-aided, digital design, and clinical case analysis are given priority, and basic research in China is relatively weak in the United States. Germany has more research but in computer software (Grayson et al., 2010; Zhou et al., 2010; Furuhata et al., 2021; Möllmann et al., 2021; Cheng et al., 2022). Regarding centrality of research, the United States, Germany, and the United Kingdom have high centrality, and the research networks of mandibular defects are mainly concentrated in Europe and North America. China lacks international cooperation despite a large number of publications.

Shanghai Jiao Tong University, the University of Sao Paulo, and the University of Michigan have the most published papers in China, Brazil, and the United States, respectively. Among the top ten institutions in the number of publications, six institutions were from Asia, indicating that treating mandibular defects has received more attention in Asia. Further analysis of institutions’ data revealed that the University of Michigan, the University of Bern, and Harvard University have the highest centrality. Institutions in the United States and Germany have close exchanges with other international institutions because of their geographical location. This indicates that institutions in the United States and Germany have more global academic influence on mandibular defects.

From the authors’ publication analysis, Wikesjoe and Ulf M. E from Georgia Regents University in the United States had the largest number of publications and the highest H-index. Their main research direction was to promote mandibular osteogenesis using the cytokine BMP-2 combined with bone tissue engineering technology (Wikesjö et al., 2008). Wei Fu-chan of Chang Gung University in Taiwan mainly published clinical studies involving vascularized free flaps for mandibular defect repair (Chang and Wei, 2021). This year, research has expanded to the mandibular allograft facial reconstruction field, which has a high academic influence (Cardona et al., 2019; De Paz et al., 2021).

### Knowledge base

Dual-MAP analysis depicted that Dentistry, Dermatology, and Surgery were the main fields of mandibular defect research, followed by molecular, biological, and immunological studies. The Journal of Craniofacial Surgery had the most publications (268); However, the Journal of Oral and Maxillofacial Surgery had the highest number of citations and H-index. This shows that the research on mandibular defects in this journal was more convincing and favored by most researchers. The most cited articles were basic research papers on stem cells, bioengineering, gene therapy, and other aspects, as well as clinical research papers on free flap reconstruction of the mandible and computer-assisted virtual surgery (Park et al., 2003; Tee and Sun, 2020; Chang and Wei, 2021; Soh et al., 2022). Dual-MAP results have attracted attention for the free flap repair of mandibular defects and stem cell tissue engineering technology.

Since the 1980s, Swartz et al. (1986), Hidalgo (1989), and Urken et al. (1989); (Swartz et al., 1986; Hidalgo, 1989; Urken et al., 1989) have reported the use of scapular flap, fibular flap, and iliac crest FLPA, respectively, for the reconstruction of mandibular defects. Free-flap reconstruction of the mandible has gradually become a mainstream method for treating mandibular defects. After more than 20 years of development, the vascularized fibular flap has become the main flap for mandibular reconstruction since 2000 (de Vicente et al., 2022; Soh et al., 2022). Recently, with the promotion of reconstructive functional surgery, the research direction of mandibular reconstruction with fibular flap has also been developed to accurately repair facial shape and stomatognathic function. Roser et al. (2010) demonstrated that a 3D printing model and osteotomy guide plate made by Virtual Surgical Panning (VSP) could improve the accuracy of mandibular repair and achieve a better repair effect than the traditional method. May et al. (2021) studied the prognosis of 264 patients who underwent mandibular reconstruction using a fibular flap. The flap-related complications of VSP operation were significantly reduced compared with those of the traditional method. Owing to the difference in height and width between the fibula and mandible, dentition restoration may be difficult. After distraction, osteogenesis was used to draw the fibula to form more bone, and the stretched fibula was used to repair the mandible, resulting in improved facial appearance and dentition restoration (Purnell et al., 2022).

Stem cell tissue engineering is a type of regenerative medicine, that is, used to treat mandibular defects. Stem cells mainly carry tissue-engineering materials to repair and replace defective mandibles (Walmsley et al., 2016). In stem cell tissue engineering, d'Aquino et al. (2009) discovered that pulp stem/progenitor cells loaded with a collagen sponge can repair several mandibular defects after third molar extraction. Mehrabani et al. (2018) compared fibrin glue scaffolds associated with adipose-derived stem cells to autologous bone grafts, and the results demonstrated that the fibrin glue scaffold associated with adipose-derived stem cells increased the thickness of new bone cortex formation and accelerated healing time. Roskies et al. (2017) used 3D-printed polyetheretherketone (PEEK) material equipped with mesenchymal stem cells to repair rabbit mandibular defects combined with distraction osteogenesis technology, achieving good surgical results. Gjerde et al. (2018) used biphasic calcium phosphate particles as a scaffold for stem cell implantation on resorbed alveolar crest, which induced new bone formation and obtained good osteogenic results. In summary, stem cell tissue engineering technology has made great progress in mandibular defect treatment; however, it is primarily used for critical mandibular defects or continuous normal mandibular defects and has not been applied to large-scale mandibular defects.

### Research hotspot and frontiers

The cluster analysis of high-frequency keywords and high-frequency cited literature can identify hotspots and preambles in the field of mandibular defect research. The top 50 keywords appear at least 80 times. Bone regeneration, surgery, and grafts are research hotspots in mandibular defects.

The latest research results have focused on the direction of tissue engineering by searching for bone regeneration. Ye et al. (2022) constructed a hierarchical vascularized engineered bone that resembled the structure of the mandible. The hydrogel was loaded with seed cells to make the bone rise again, and a good repair effect on large mandibular defects was achieved.

Stevanovic et al. (2022) compare hydroxyapatite/poly real (lactide-co-glycolide) and hydroxyapatite/polyethyleneimine composite scaffolds. Hydroxyapatite/polyethyleneimine composite scaffolds carrying allograft can obtain a better effect on bone regeneration. Similar to surgery and grafts, 3D-printed and virtual surgical planning (VSP) research on mandibular defect repair with free flaps have attracted the most attention. Steybe et al. (2022) analyzed the computer-aided design of a deep circumflex iliac artery flap for mandibular defects to improve the accuracy of mandibular defect repair. Nyirjesy et al. (2022) summarized the application of CAD/CAM and 3D-printed in reconstructing defects after head and neck tumor defects, which can significantly reduce the operation time and improve the surgical effect.

Based on the analysis of the co-cited literature and combined with recent studies, we speculate that the oral function and dentition precise repair of mandibular defects, the reconstruction of chewing and swallowing functions through computer-aided design, and 3D printing osteotomy guide plate may be the ongoing research hotspots in the direction of mandibular defects (Bak et al., 2010; Foley et al., 2013; Nyirjesy et al., 2022). Traditional methods are becoming increasingly difficult to meet the requirements of precise mandibular reconstruction because of the 3D anatomical structure and complex function of the face. The latest research mentioned the use of biomaterials for mandibular defects, allogeneic transplantation of facial tissue, and drug-induced rapid distraction osteogenesis (De Paz et al., 2021; Al Maruf et al., 2022; Shen et al., 2022), providing a new idea for the study of mandibular defects. In summary, combined with the bibliometric analysis, among the various methods of reconstruction in the field of mandibular defects, we consider that accurate mandibular reconstruction and tissue engineering bone regeneration technologies are research hotspots and frontiers in this field.

## Limitations

Our study has some limitations. Literature metrology is the most commonly used database in WoSCC. However, if comprehensive analysis is required, Scopus or PubMed databases and existing metrology research-related software must be considered. Additionally, this study only considered English literature, and the relevant literature in 2022 was excluded, and we may have missed some research hotspots.

## Conclusion

1. Over the past 20 years, the number of studies on mandibular defects has gradually increased.
2. After more than 20 years of development, new surgical procedures are increasingly being used in clinical practice.
3. The United States, Germany, and China contribute to mandibular defect research.
4. Computer-aided design, 3D printing osteotomy and reconstruction guide plates, virtual surgical planning, and bone tissue engineering will be the focus of future research.
5. This study systematically analyzed the relevant literature on mandibular defects and reported research results from 2000 to 2021, laying a foundation for further in-depth research on mandibular defects worldwide.

## Data Availability

The original contributions presented in the study are included in the article/supplementary material, further inquiries can be directed to the corresponding author.
